# An Enhanced MEMS Error Modeling Approach Based on Nu-Support Vector Regression

**DOI:** 10.3390/s120709448

**Published:** 2012-07-09

**Authors:** Deepak Bhatt, Priyanka Aggarwal, Prabir Bhattacharya, Vijay Devabhaktuni

**Affiliations:** 1 EECS Department, The University of Toledo, MS 308, 2801 W. Bancroft St., Toledo, OH 43606, USA; E-Mails: Priyanka.Aggarwal@utoledo.edu (P.A.); Vijay.Devabhaktuni@utoledo.edu (V.D.); 2 School of Computing Sciences & Informatics, University of Cincinnati, 814B Rhodes Hall, Cincinnati, OH 45221, USA; E-Mail: bhattapr@ucmail.uc.edu

**Keywords:** MEMS IMU, Neural Network, Support Vector Machines

## Abstract

Micro Electro Mechanical System (MEMS)-based inertial sensors have made possible the development of a civilian land vehicle navigation system by offering a low-cost solution. However, the accurate modeling of the MEMS sensor errors is one of the most challenging tasks in the design of low-cost navigation systems. These sensors exhibit significant errors like biases, drift, noises; which are negligible for higher grade units. Different conventional techniques utilizing the Gauss Markov model and neural network method have been previously utilized to model the errors. However, Gauss Markov model works unsatisfactorily in the case of MEMS units due to the presence of high inherent sensor errors. On the other hand, modeling the random drift utilizing Neural Network (NN) is time consuming, thereby affecting its real-time implementation. We overcome these existing drawbacks by developing an enhanced Support Vector Machine (SVM) based error model. Unlike NN, SVMs do not suffer from local minimisation or over-fitting problems and delivers a reliable global solution. Experimental results proved that the proposed SVM approach reduced the noise standard deviation by 10–35% for gyroscopes and 61–76% for accelerometers. Further, positional error drifts under static conditions improved by 41% and 80% in comparison to NN and GM approaches.

## 1. Introduction

Micro Electro Mechanical System (MEMS)-based inertial sensors (accelerometers and gyroscopes) have been embraced by the auto industry in their quest to improve performance, reduce cost and to enhance the reliability of the vehicles [1]. MEMS have enabled the sensor technology to evolve from restricted, expensive, and inflexible units to miniaturized, low-cost and low-power silicon-based units [2]. Although, being small in size and light in weight, MEMS sensors experience more errors like turn-on to turn-on biases, in-run biases, scale factor drifts and other environment dependent errors, which are generally small or negligible for higher grade sensors [3,4]. These errors build up over time, corrupting the precision of the measurements and rendering the navigation solution useless. For example, a higher grade IMU with a gyroscope bias of 1 °/h will experience a position error of 1.7 m in a minute, while a MEMS IMU with gyroscope bias of 100 °/h will have an error of 171 m. Therefore, for seamless and continuous navigation solution from MEMS sensors, the modeling of errors and their reliable estimation or compensation is mandatory. We solve this challenge by developing advance error models based on support vector machines.

The inertial sensor errors are divided into two main parts: systematic/deterministic and dynamic/random. The deterministic error sources mainly include the biases and the scale factor errors, which remain constant during a run and can be removed by specific calibration procedures in a laboratory environment [3]. The detailed laboratory calibration process through six-position static testing, multi-position static testing and angular rate testing have been explained by number of researchers [3–6]. However, for low-cost MEMS sensors, these systematic errors are quite large and their repeatability is typically poor because of their environmental dependence (especially temperature) which makes frequent calibration a necessity [5,6]. In view of these facts, extensive temperature-dependent modeling of the bias and scale factor errors is investigated [7]. The random or stochastic part of inertial sensor errors can be attributed to random noise in the signals, leading to random variations or drifts in bias or scale factor over time. These random noises consist of low frequency (long-term) component and a high frequency (short-term) component [8]. The high frequency component has white noise characteristics while the low frequency component is characterized by correlated noise and causes gradual change in errors during a run. A wavelet de-noising technique is generally used to remove the high frequency component while the lower frequency component is stochastically modeled [8]. There are number of stochastic or random processes available for modeling these slowing drifting biases and scale factor errors such as random constant, random walk, Gauss Markov (GM) process and Auto Regressive (AR) model [9–14]. Usually, these processes exploit the autocorrelation or Allan variance function of the noise to obtain first-order GM or other higher order auto-regressive model parameters [8]. The value of the random walk parameters can be determined from the standard deviation of a sufficiently long static data, through correlation between values of the noise at different points in time (autocorrelation process) or by representing root-mean-square drift error as a function of averaged time (Allan variance technique) [8].

Thus, before deploying MEMS based accelerometers and gyroscopes for vehicular navigation, an accurate determination of systematic and random errors is required to ensure the acceptable performance. The deterministic errors can be estimated using different lab calibration procedures as explained in [3–8], which are then removed from the raw measurements. However, modeling the random errors accurately is often a difficult task due to the presence of noises in the sensor measurements. Traditional approaches of random error modeling like GM model and Allan variance method work unsatisfactorily for MEMS sensors [14]. Moreover, the whole process of modeling the static and dynamic biases are extremely complex and sometimes do not provide the reliable estimates, thereby affecting the navigation accuracy of the system. Alternatively, artificial intelligence approaches utilizing Neural Network (NN) have been utilized in modeling the MEMS error and are found to perform better than other conventional techniques [14,15]. However in this particular case, NN suffers from poor generalization capability due to the presence of an elevated level of noises in the input-output data to be modeled. Hence, the NN model prediction accuracy is poor and deteriorates after a short time. Also, the model development process takes longer time, which limits their real-time implementation [16]. Recently, Support Vector Machines (SVMs) based techniques have been applied to model the MEMS error [17,18]. Support Vector Machines (SVMs) based on the structural risk minimization principle can avoid local minimization and over-fitting problems as encountered in NN, thus improving the prediction accuracy. As opposed to neural networks, it requires less training time, and hence is suitable for real-time implementation. This paper thus proposes the implementation of an enhanced Nu-Support Vector Regression (Nu-SVR) technique for modeling these random and substantial MEMS sensor errors [19]. The proposed approach is different from those presented in [17,18], as it automatically selects the model parameter (*i.e.*, error margin), and the priori knowledge of the noise model is not mandatory [20]. Like the NN approach, the Nu-SVR model utilizes the same set of input-output sample pairs to model the errors. Once the Nu-SVR model is trained, it is utilized to predict the desirable output over an independent set of sample pairs. To test the efficacy of the proposed model, a low-cost MEMS Inertial Measurement Unit (IMU) manufactured by Cloud Cap Technology known as Crista IMU is employed [9].

The paper has been divided into five sections. Section 2 covers the conventional approaches of modeling the MEMS sensor errors. Section 3 explains the working of support vector regression. Experimental setup and the calibration results obtained using conventional and the proposed approaches are detailed in Section 4, along with their impact on the navigation solution accuracy. Finally, Section 5 concludes the paper.

## 2. Conventional Error Modeling Approaches

There are numbers of errors like bias, scale factor, cross-axis sensitivity or misalignment, noise and temperature drifts that affect the performance of inertial sensors [5]. Bias is the output observed when no input is applied. It can be divided into two parts, namely, static bias and dynamic bias. The static portion consists of fixed bias (which is calibrated and removed by a six-position static test method), turn-on to turn-on bias which is a small change in bias with each run [4,5] and bias variation due to change in temperature. The dynamic portion consists of remaining biases that could not be compensated and consist of random low and high frequency noises. The high frequency component has white noise characteristic while the low frequency component is characterized by a correlated noise. As the manufacturer has calibrated and reasonably compensated offset bias based on a series of undisclosed tests [10], the effect of rest of the bias components is evaluated as given in Equation (1).

(1) $$b=b_{\text{turn}-on}+b_{\text{temp}}+b_{in-\text{run}},$$

where *b_turn-on_* is the turn-on to turn-on bias, *b_temp_* is the temperature dependent bias while *b_in-run_* is the in-run bias.

The scale factor is the ratio of a change in output to a change in the intended input to be measured [3]. The scale factor error for the Crista IMU has been calibrated and compensated by the manufacturer and is considerably smaller than turn-on bias. Cross-axis sensitivity errors are caused by misalignments between the axes of sensor triads which should ideally be placed orthogonal to each other. Again, as the manufacturer has compensated major portions of the deterministic errors, the emphasis mainly lies on estimating the remaining biases (turn-on to turn-on, in-run) and noises.

Thus, the static portion consisting of turn-on to turn-on biases, temperature dependent biases and a deterministic portion of in-run biases are evaluated using specific lab calibration procedure as discussed later in Section 2.2, whereas the remaining portion of in-run errors (also known as random errors) are estimated using stochastic processes such as GM process and Allan variance methodology. Typically, modeling the random portion of in-run biases using the above mentioned processes involves cumbersome mathematical calculations and is often inaccurate [16]. Recently, the Radial Basis Function Neural Network (RBFNN) approach is implemented to model the MEMS random drift. The RBFNN method was effectively able to reduce the standard deviation of sensor noises compared to traditional approaches [16]. Another advantage of utilizing RBFNN is that it does not require the mathematical knowledge of the various noises involved and models them using the given input-output sample pairs. The details of the low-cost MEMS Crista IMU considered in this study are discussed next.

### 2.1. Manufacturers Specifications

#### 2.1.1. Crista IMU

Crista inertial sensor is a small 3-axis MEMS device that consists of three single-axis ADXRS 610 Analog devices gyroscopes and a tri-axis KXD94-2802 accelerometer by Kionix [9]. This is a small unit that provides both high resolution raw angular rates and acceleration data via a serial interface (RS-232). It also provides compensated data in engineering units after removing static offsets and temperature-induced errors. The key specifications of the Crista IMU are given in Table 1.

The manufacturer passes the raw data through a low-pass filter to remove high frequency noise components before data is sampled by a 16-bit analog-to-digital converter. Die temperatures are also measured on each gyroscope and compared to a 10-point temperature calibration table stored in the memory (EEPROM) of the IMU. This in-built procedure corrects the measured sensor outputs by removing any temperature-induced errors and non-linearities. IMU also stores correction matrices to account for any misalignment errors and linear acceleration sensitivities so as to maximize its off-shelf accuracy. The user controls both the data update rate and the over-sample averaging output rate of the unit.

### 2.2. Laboratory Calibration and Modeling of Inertial Sensor Errors

The following section briefly explains the existing methods for estimating the static biases using a six-position static test method for accelerometer and a simple averaging method for gyroscopes. Thermal calibration method for quantifying the drifts under varying temperature is given next, followed by the process to evaluate GM model parameters.

#### 2.2.1. Six-Position Static Test

The six-position static and rate tests are among the most commonly used calibration methods [5]. The six-position method involves mounting the inertial system on a levelled surface with sensitive X, Y and Z axes of the IMU pointing alternately up and down. For a triad of orthogonal sensors, this results in a total of six positions. The bias and scale factor errors are calculated using the following Equations (2) and (3):

(2) $$b_{\text{fix},\text{turn}-on}=\frac{I_{f}^{up}+I_{f}^{\text{down}}}{2}$$

(3) $$S_{a}=\frac{I_{f}^{up}-I_{f}^{\text{down}}-2\times K}{2\times K}$$

where $I_{f}^{up}=b-(1+S_{a})$ is the sensor measurement when the sensitive axis is pointed upward, $I_{f}^{\text{down}}$ is the measurement when the sensitive axis is pointed downward and *K* is the known reference signal. For accelerometers, *K* is the Earth's local gravity constant and for gyroscopes, *K* is the magnitude of the earth rotation rate projection to the vertical at given latitude (for higher grade sensors). The above explained six-position static tests cannot calibrate automotive grade MEMS gyroscopes since bias instability and noise levels completely mask the earth's rotation reference signal. For low-cost MEMS gyroscopes, angular rate tests are conducted using a turn-table which is rotated at a user-defined rate. A quicker way of obtaining the biases for the gyroscope is through averaging the data for a short time duration under static conditions (say, for example, a minute), as any non-zero value for a static gyroscope mainly indicates the bias. The same principle can be used for an accelerometer if it is completely levelled and does not observe any component of Earth's gravity.

#### 2.2.2. Thermal Calibration Method

Thermal calibration is required to compensate for the thermal drifts of MEMS IMUs. There are two main approaches for thermal testing, *i.e.*, the Soak method and the Ramp method. In the Soak method, the IMU placed in a closed thermal chamber is allowed to stabilize at a particular temperature before recording the sensor data. In the Ramp method, the IMU temperature is linearly increased or decreased for a certain period of time. Here, we have used the hot air gun to observe the thermal drifts according to the Ramp method where the temperature of the IMU linearly increases without a stabilization period [21]. Inertial sensor random errors are modeled by passing white noise through shaping filters (denoising) to yield time-correlated noises after removing the high frequency component of the signal.

#### 2.2.3. Autocorrelation Process

The autocorrelation function of a discrete signal is the product of the random signal with a time-shifted version of itself. Therefore, it is widely used for characterization of correlated and slowly drifting noises. If *x*(*k*) is a random sequence, its autocorrelation function is given by Equation (4) [22–25]:

(4) $$R_{x}(\tau )=E(x(k)x(k+\tau ))$$

where *E* is the expectation operator and ***τ*** is the time shift. The autocorrelation function of a white noise has zero correlation for all lags other than zero and has a dirac delta function (δ) as its value at zero. This means that white noise has infinite variance, which is realistically not feasible. Hence, to account for the effect of white noise, numbers of random processes are generated by passing white noise through shaping filters. A commonly used random process to model slowly drifting bias (*i.e.*, in-run bias) is the GM process. Its exponential autocorrelation function is given by Equation (5):

(5) $$R_{x}(\tau )=\sigma^{2}e^{-\beta |t|}$$

where *β* is the inverse of the correlation time (1/e point) and *σ* is the calculated standard deviation [22]. In continuous time domain, the first-order Gauss Markov process is given by Equation (6):

(6) $$\dot{x}(t)=-\beta x(t)+\sqrt{2\sigma^{2}\beta }w(t)$$

On applying forward Euler integration, Equation (9) is obtained:

(7) $$\frac{dx(t)}{dt}=\frac{x_{k+1}-x_{k}}{\Delta t}$$

Therefore:

(8) $$\frac{x_{k+1}-x_{k}}{\Delta t}=-\beta x_{k}+\sqrt{2\sigma^{2}\beta }w_{k}$$

(9) $$x_{k+1}=(I-\beta \Delta t)x_{k}+\sqrt{2\sigma^{2}\beta }w_{k}\Delta t$$

Random walk is another stochastic process obtained when white noise is integrated. In a mechanization process (a process of converting gyroscope and accelerometer data into navigation parameters), both gyroscope and accelerometer signals, which contains white noise components, are integrated to obtain change in angles and velocities. Hence, angles and velocities are corrupted with these integrated white noise components, called angular random walk and velocity random walk, which are usually obtained through the Allan variance method.

#### 2.2.4. Allan Variance Methodology

Allan variance is a method of representing root mean square random drift error as a function of averaged time [26]. The Allan variance, first introduced as a means to quantify the precision of atomic oscillators, can be used to identify all parameters necessary to fully model and emulate sensor errors. The Allan variance provides a means of quantifying the various stochastically driven error sources present on an inertial sensor output. The data are plotted as the square root of the Allan variance verses T (time) on a log-log plot [21,27,28]. It is simple to compute and relatively easy to interpret and understand as different slopes on the log-log plot indicate the various dominating noises present for the particular sensor.

For very low-cost MEMS IMUs, we are mainly concerned with the noise terms whose correlation time is much shorter than the sample time and contribute to the gyroscope Angle Random Walk (ARW) and accelerometer Velocity Random Walk (VRW) noise components, along with slowly drifting in-run biases. The values for ARW and VRW are obtained from (10):

(10) $$\sigma (T)=\frac{Q}{\sqrt{T}}$$

where *Q* is the angle (velocity) random walk coefficient and each cluster has time *T*, which is equal to collection of *n* data samples. It is evident from Equation (7) that a log-log plot of *σ*(*T*) *vs*. T has a slope of −1/2. Thus, a numerical value of Q can be obtained directly by reading the slope line at T = 1 s. These deterministic and random error sources present in low-cost IMU (*i.e.*, Crista IMU) are evaluated in Section 4.1. Another existing approach (*i.e.*, RBFNN) to model low-cost IMU error is explained next.

### 2.3. Modeling Random Errors Using RBFNN

RBFNN, one of the artificial intelligence approaches, is used for modeling the functional relationship corresponding to the given input-output sample pairs [29,30]. Similar to a three layer multilayer perceptron neural network, it has three layers known as input, hidden and output layers. The input layer neurons are relay neurons, *i.e.*, it simply passes the input data to the next layer. However, the net input to the hidden layer neurons (also known as radial basis neurons) is the vector distance between its input vector and the weight vector, multiplied by the associated hidden neuron biases. The transfer function for hidden layer neurons is:

(11) $$f(n)=e^{-n^{2}}$$

where *n* represents the inputs to the hidden layer neurons. The activation function of each neuron in the output layer is linear and thus outputs the weighted sum of the hidden layer neurons output with an addition of bias.

To model the random drift using existing RBFNN, we make use of the autocorrelation property. According to this property, the measurement system output at time instant *n* is dependent on its adjacent and near-adjacent measurement. Therefore, the output at any time instant *n* can be represented as a function of previous *n* − 1 outputs:

(12) $$x_{n}=f(x_{1},x_{2},\ldots ,x_{n-1})$$

Thus, the RBFNN models the functional relationship using the input-output sample pairs as depicted by Equation (12). The developed functional relationship can be then utilized to predict the sensor's output *x*ˆ*_n_* at time instant *n*. The difference between the predicted output and the actual output (*x_n_* − *x*ˆ*_n_*) is termed as residuals. However, for MEMS sensors, the performance of RBFNN is limited since the data is highly complex with substantial amount of noise. As a result, RBFNN model suffers from poor generalization capability, increased training time, and poor prediction accuracy even for a short duration data. We overcome these challenges of the limited RBFNN model performance by implementing sophisticated models based on Nu-SVR method.

## 3. Proposed Nu-SVR Methodology

Support vector machines as described in [31] have shown to deliver a promising solution in various classification and regression tasks due to its ability to avoid local minima, improved generalization capability, and sparse representation of the solution. Unlike NNs, which are based on Empirical Risk Minimization principle (ERM), SVMs are based on the Structural Risk Minimization (SRM) principle and thus try to control the upper bound of generalization risk while reducing the model complexity.

### 3.1. Nu-SVR Principle

Given a set of input-output sample pairs {(***x*_1_, *y*_1_**), (***x*_2_, *y*_2_**),…, (***x***_n_, ***y***_n_)} the objective of Nu-SVR technique is to approximate the nonlinear relationship given in Equation (13), such that *f*(*x*) should be as close as possible to the target value ***y*** and should be as flat as possible in order to avoid over-fitting:

(13) $$f(x)={\text{w}}^{T}.\Phi (x)+b.$$

In Equation (13)***w****^T^* is the weight vector, *b* is the bias and Φ(*x*) represents the non-linear mapping function which maps the input space to a higher dimensional space. To ensure that the approximated function meets the above two objectives of closeness and flatness, the primal objective of the problem is to minimize:

(14a) $$\frac{1}{2}{\left\|\text{w}\right\|}^{2}+C\left\{ϒ.ɛ+\frac{1}{n}\sum_{i=1}^{n}\left(\xi +\xi^{*}\right)\right\};$$

subject to the constraints:

(14b) $$\begin{array}{l} y_{i}-\langle {\text{w}}^{T}.\Phi \left(x\right)\rangle -b\leq ɛ+\xi_{i}^{*},\\ \langle {\text{w}}^{T}.\Phi \left(x\right)\rangle +b-y_{i}\leq ɛ+\xi_{i},\\ \xi_{i}^{*},\xi_{i}\geq 0. \end{array}$$

where *ε* is a deviation of a function *f*(*x*) from its actual value, and *ξ*, ξ* *i* are additional slack variables introduced by cortex and vapnik in [32], which determines that deviations of magnitude *ξ* above *ε* error are tolerated. The constant C, known as the regularization parameter, determines the tradeoff between the flatness of *f* and tolerance of error above *ε*. Further ϒ (0 ≤ ϒ ≤ 1) represents the upper bound on the function of margin errors in the training set and establishes the lower bound on the fraction of support vectors. To solve the primal problem in Equation (14a), its dual formulation is introduced by constructing Lagrange function (*L*) given as:

(15) $$L:\frac{1}{2}{\left\|\text{w}\right\|}^{2}+C\left\{ϒ.ɛ+\frac{1}{n}\sum_{i=1}^{n}\left(\xi +\xi^{*}\right)\right\}-\frac{1}{n}\sum_{i=1}^{n}\left(\eta .\xi +\eta^{*}.\xi^{*}\right)-\frac{1}{n}\sum_{i=1}^{n}\left(ɛ+\xi_{i}+y_{i}-{\text{w}}^{T}.\Phi \left(x\right)-b\right)-\frac{1}{n}\sum_{i=1}^{n}\left(ɛ+\xi_{i}-y_{i}+{\text{w}}^{T}.\Phi \left(x\right)+b\right)-\beta .ɛ.$$

where *α*, α*, *η*, η*, *β* are Lagrange multipliers and *α*^(^*^)^ = α.α*. Thus, maximizing the Lagrange function gives $w=\overset{n}{\underset{i=1}{\text{∑}}}\left(\alpha_{i}-\alpha_{i}^{*}).\Phi (x_{i}\right)$ and yields the dual optimization problem:

(16a) $$\text{maximizes}-\frac{1}{2}\sum_{i,j=1}^{n}\left(\alpha_{i}-\alpha_{i}^{*}\right).(\alpha_{j}-\alpha_{j}^{*}).K(x_{i},x_{j})+\sum_{i=1}^{n}y_{i}.(\alpha_{i}-\alpha_{i}^{*});$$

subject to:

(16b) $$\begin{array}{l} \sum_{i=1}^{n}\left(\alpha_{i}-\alpha_{i}^{*}\right)=0,\\ \sum_{i=1}^{n}\left(\alpha_{i}+\alpha_{i}^{*}\right)\leq Cϒ,\\ \alpha_{i},\alpha_{i}^{*}\in [o,\frac{C}{n}]. \end{array}$$

where *K*(*x_i_*,*x_j_*) denotes the kernel function given by *K*(*x_i_*,*x_j_*) = Φ(*x_i_*)*^T^*.Φ(*x_j_*). The solution to Equation (16a) yields the Lagrange multipliers *α*, α*. Substituting weight *W* in Equation (13), the approximated function is given as:

(17) $$f(x)=\sum_{i=1}^{n}\left(\alpha_{i}-\alpha_{i}^{*}\right).K(x_{i},x)+b.$$

Usually there are four types of kernels used, namely, polynomial function, Radial Basis Function (RBF), sigmoid function and linear function. Selecting an appropriate kernel for a given problem improves the model prediction accuracy. In our study we selected an RBF kernel, as it delivers an acceptable accuracy and has less implementation difficulties [33]. The parameter *b* is identified using Karush-Kuhn-Tucker conditions [34,35]. For further details related to Nu-SVR working, please refer to [20,36].

Thus, given input-output training sample pairs, the Nu-SVR approach identifies the Lagrange multipliers *α*, α* and *b*. After parameter identification, the model can be utilized to predict the output corresponding to an unknown input using Equation (17).

## 4. Experimental Setup and Results

Laboratory tests were conducted on static data to identify the various error terms for Crista IMU. The manufacturer has already removed the fixed bias, temperature-induced errors and non-linearities from the raw IMU data and therefore they are not taken into account during MEMS error modeling. To model the remaining errors, three different approaches are considered in this paper which includes two traditional approaches: (a) determining static and dynamic biases through specific lab calibration procedures (as explained in Section 2.2.1–2.2.4); (b) An RBFNN method as described in Section 2.3; and (c) lastly, the proposed Nu-SVR approach. The detailed experimental processes and results of MEMS error modeling using the above three processes are described next.

### 4.1. Evaluating Static and Dynamic Biases Using In-Lab Calibration Procedures

**Figure 1.**
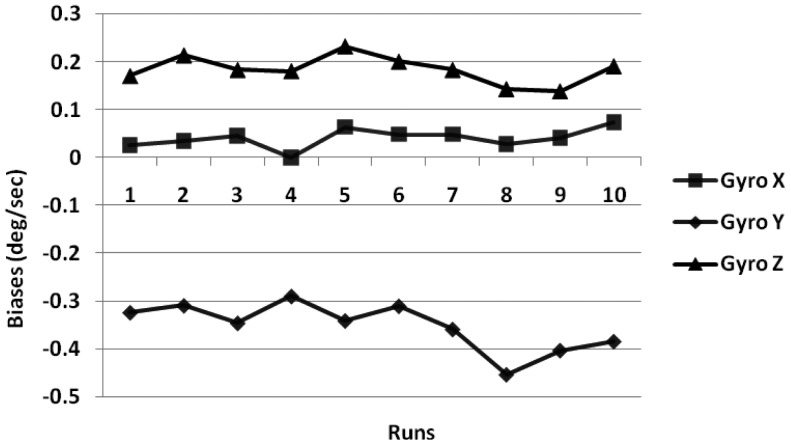
Gyroscope turn-on to turn-on biases.

We performed laboratory calibration of all the three gyroscopes and three accelerometers for low-cost Crista IMU. The turn-on to turn-on biases were calculated by averaging the static data collected for 1 minute for the gyroscopes (at 85 Hz) under stabilized room temperature for 10 runs taken at different times, over a period of 10 days (Figure 1).

**Figure 2.**
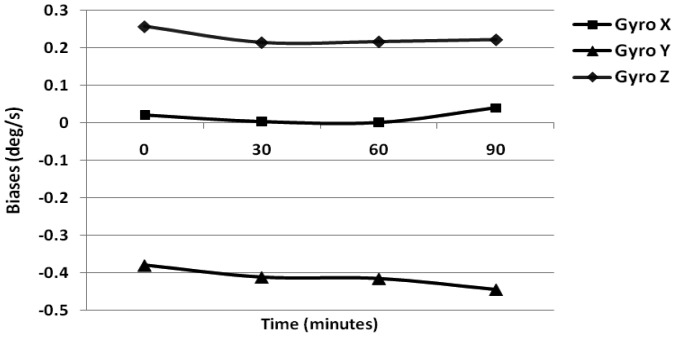
Gyroscope in-run biases (const. temp.).

The turn-on to turn-on bias obtained for the gyroscope and accelerometer is 0.23 °/s and 0.12 m/s^2^. Next the in-run biases were calculated using 1 min of static data collected after 30 min intervals over a 90 min duration, when the IMU temperature had stabilized. The results obtained using three gyroscopes are shown in Figure 2.

For gyroscopes, simple averaging was performed for 1 min of data while for accelerometers, the six-position static test method was incorporated. The in-run bias obtained for gyroscope is 0.25 °/s while that specified by the manufacturer is less than 0.20 °/s. Similarly, for the accelerometer the obtained average value is 0.12 m/s^2^, which is higher than the manufacturer value of 0.02 m/s^2^ at constant temperature. Further, to obtain the in-run biases under varying temperature conditions, we heated the IMU continuously using a heat gun (including a warm-up period). The internal temperature of the Crista IMU is observed for 1.5 h including the warm-up period where the temperature of the IMU continuously increases as per the Ramp method.

**Figure 3.**
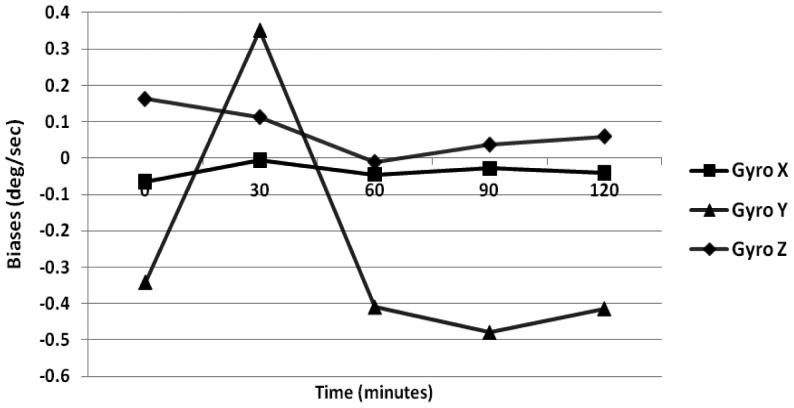
Gyroscope in-run biases (vary temp.)

The temperature of the IMU is allowed to change from 20 °C to 75 °C which is the operating temperature range of the Crista IMU. The results obtained for the gyroscope drifts are illustrated in Figure 3. The maximum bias variation for the gyroscope is 0.35 °/s, and that of the accelerometer is 0.15 m/s^2^. Further, it is illustrated in Figure 3 that the bias variations are high only within the first 30 min (which is the heating period of IMU). Next, to model the dynamic portion of the biases Allan variance method and GM parameter are estimated.

#### 4.1.1. ARW and VRW Parameters by Allan Variance Method

**Figure 4.**
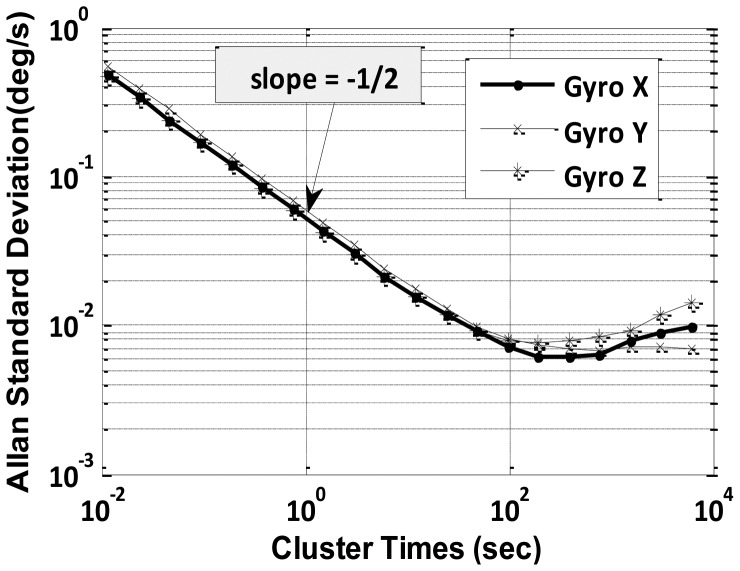
Allan variance for gyroscopes.

**Figure 5.**
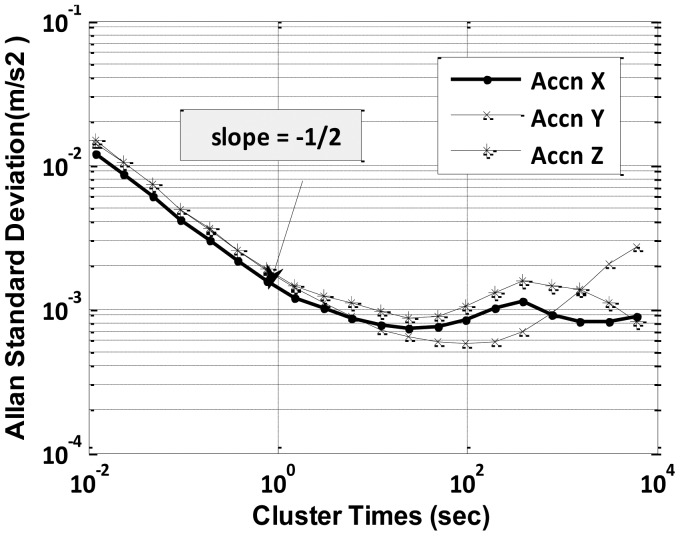
Allan variance for accelerometers.

Static data was collected for 12 h and was processed by Allan variance method to obtain velocity and angular random walk parameters. A log-log plot of *σ*(*T*) *vs. T* has a slope of −1/2 as indicated in Equation (10). The numerical value of *Q* is obtained directly by reading the slope line at *T* = 1 s from Figures 4 and 5.

The initial downward slope of −1/2 indicates the IMU's primary error source is angular or velocity random walk as hypothesized. Further, effects of temperature changes on biases of gyroscope and accelerometer were determined by running the IMU continuously for a period of 12 h while the heat gun was occasionally used to vary the temperature of the IMU, including the warm-up period. The obtained ARW and VRW parameters are given in Table 2.

#### 4.1.2. Gauss Markov Model Parameters by Autocorrelation and Allan Variance Method

**Figure 6.**
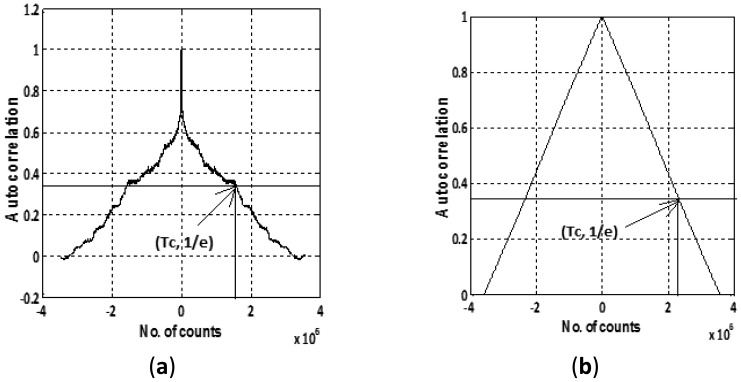
Autocorrelation sequence: (**a**) gyroscope; (**b**) accelerometer.

IMU sensor errors are generally modeled by first-order Gauss Markov process that requires two parameters, *i.e.*, correlation time and standard deviation as stated by Equation (9). To obtain these two essential parameters, we collected static data for 12 h which was first denoised to remove the effect of high frequency noise component. Figure 6 shows the plot of the autocorrelation sequences for two of the Crista sensors, namely, gyroscope X and accelerometer X.

The correlation time (in sec) for each of the gyroscope and accelerometer is calculated by dividing the number of counts (*T_c_*) with the sampling frequency (85 Hz) corresponding to the value equal to 1/e on the y axis as given in Table 3. The standard deviation in Table 3 is calculated by observing the average deviation in static data after denoising. These evaluated deviations are further verified by the Allan variance plots (Figures 4,5) by reading the value for bias instability corresponding to slope of 0 degrees. The laboratory calibration shows that except turn-on to turn-on and in-run bias at varying temperature, the other types of errors, *i.e.*, in-run bias (at constant temp) and ARW, VRW values are not within the manufacturer specified range.

### 4.2. Error Modeling Using RBFNN

A total of 31,100 static IMU data was collected for duration of 6 min at a sampling frequency of 85 Hz. The collected dataset is then labeled as [*x*_1_, *x*_2_, *x*_3_,…, *x*_31100_]. A part of the collected dataset is used to build the model and the rest is used to test the model. The matrix of training input is defined as [*T*_1_, *T*_2_, *T*_3_,…, *T_m_*], where *T_i_* = [*x_i_*, *x_i_*_+1_, *x_i_*_+2_,…, *x_i_*_+_*_n_*_−1_]*^T^*, and *I* = 1,2,…,*m*. The vector of desired training output is represented as [*x_n_*_+1_, *x_n_*_+2_, *x_n_*_+3_,…,*x_n_*_+_*_m_*]*^T^*. To identify the suitable value of inputs (*n*), different models for each inertial sensor (3 gyroscopes and 3 accelerometers) with varying values of *n* were developed and tested to check the model performance. Table 4 shows the variation in the training time and the standard deviation of the compensated and raw data with the number of inputs for one of the model (*i.e.*, gyroscope X). Similar results were observed for the other models. Hence it was identified that *n* = 10 shows the best tradeoff between least standard deviation and the training time. Out of a total of 31,100 samples, first 500 samples were utilized to train the RBFNN model. The model was trained using MATLAB code (Mathworks, Inc., Natick, MA, USA), and the training error set to 0.001.

**Figure 7.**
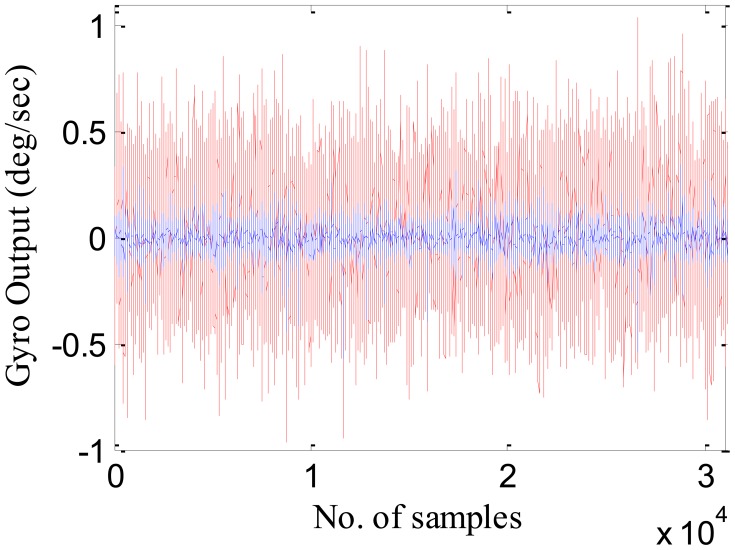
Gyroscope X output, red: uncompensated, blue: compensated.

The trained model was then tested using an independent set of data known as the testing set. Figure 7 shows the gyroscope X actual (in red) and the error compensated output (in blue) using the RBFNN model. Table 5 shows the standard deviation of the raw data and the compensated data set after applying this formulated RBFNN model.

As shown in Table 5, the standard deviation of noise in the compensated dataset has been considerably reduced in comparison to raw IMU measurements. This noise reduction will further reduce the positional errors, leading to seamless navigation.

### 4.3. Error Modeling Using Proposed Nu-SVR Approach

Corresponding to the RBFNN approach, six Nu-SVR models are developed to model the random errors associated with each of the six inertial sensors. For training the Nu-SVR model, the data is arranged in a similar pattern as described in Section 4.2, with the training input and the output vector defined as *T_i_* = [*x_i_*, *x_i_*_+1_, *x_i_*_+2_,…, *x_i_*_+_*_n_*_−1_]*^T^* and [*x_n_*_+1_, *x_n_*_+2_, *x_n_*_+3_,…, *x_n_*_+_*_m_*]*^T^* respectively. During the testing phase, this trained Nu-SVR model is utilized to compensate for the MEMS sensor errors. However, the model accuracy depends on the appropriate selection of the kernel function and the regularization parameter (C). Out of the different types of kernel functions, the commonly used radial basis function (RBF), with parameter gamma (γ), was selected because of its faster training rate and less implementation difficulty as given in Equation (18). In the training phase, optimal values for C and gamma parameters are obtained for each of the inertial sensor as depicted in Table 6.

(18) $$K(x_{i},x)=\exp\left\{-\gamma {\left|x_{i}-x\right|}^{2}\right\}.$$

The accuracy of these trained models using the identified model parameters are then evaluated using the independent set of data known as the testing set. The Nu-SVR technique in this paper is implemented using LibSVM software [37]. Table 7 shows the standard deviation of the raw datasets and that of the compensated datasets using the proposed Nu-SVR model.

**Figure 8.**
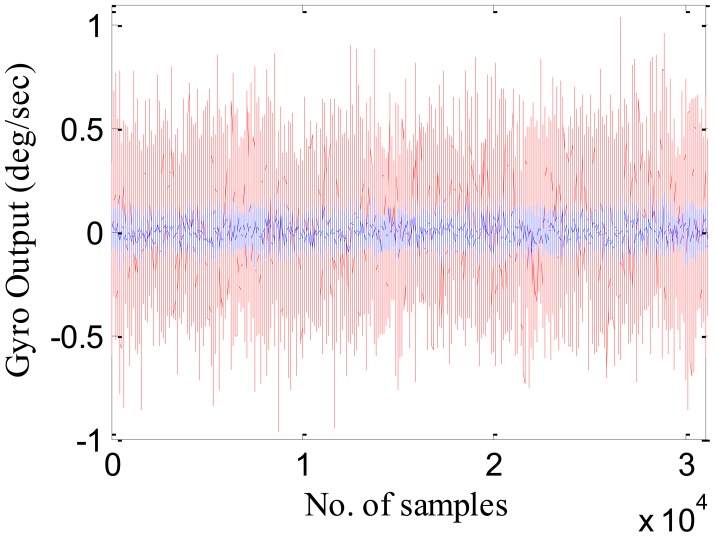
Gyroscope X output, red: uncompensated; blue: compensated using Nu-SVR approach.

Figure 8 shows the gyroscope X actual (in red) and the compensated output (in blue). It is evident from the figure that compensated output or the residuals have less deviation than the actual output.

**Figure 9.**
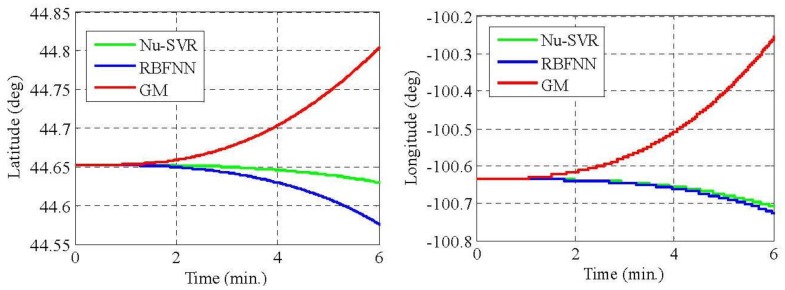
Position drifts by GM, RBFNN and Nu-SVR methods.

**Figure 10.**
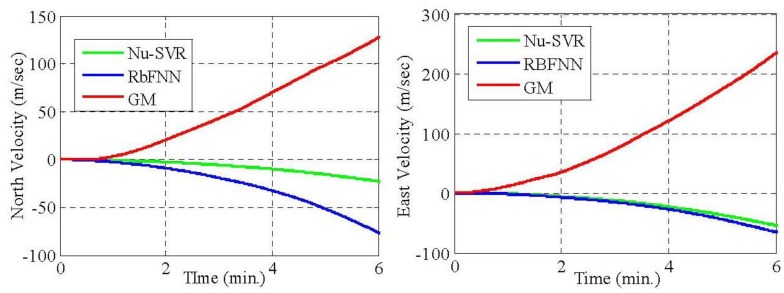
Velocity drifts by GM, RBFNN and Nu-SVR approaches.

From Tables 5 and 7, it is evident that the proposed approach of modeling MEMS error using Nu-SVR is faster and more accurate than the RBFNN method. For each of the three gyroscopes, the training time speeds up 10 times whereas for accelerometers it is almost half. Also, the maximum percentage improvement in the reduction of standard deviation when compared to RBFNN method is found to be 76%. Thus, the result shows that the proposed Nu-SVR method of modeling MEMS error is much more accurate and much faster. Also, unlike the modeling process explained in Section 2.2, the proposed approach is quite simple and fast and does not require the tedious lab calibration process. To further compare the performance of Nu-SVR methodology, drifts in position solution were obtained by applying mechanization equations for all the methods as illustrated in Figures 9 and 10.

For the GM model, IMU raw data compensation is carried out using the parameters identified in Section 4.1. Ideally, since the IMU is static, the drifts in the positional and velocity components should be zero. Any deviation from its original value shows the presence of errors. Thus larger drift reflects ineffectiveness of the approach in compensating the errors. It is clearly illustrated in Figures 9 and 10 that the positional and velocity drifts obtained using our proposed Nu-SVR approach is much smaller than the two existing methods. The percentage improvement in the positional accuracy is 41% and 80% when compared to RBFNN and GM approaches, respectively, under static conditions. It should be noted that this is a preliminary research employing a Nu-SVR technique for modeling MEMS sensor errors. In our future work we will illustrate the performance of the Nu-SVR algorithm in dynamic environment.

## 5. Conclusions

MEMS sensors are lightweight and low-cost but have large errors as compared to other higher grade inertial sensors. This paper introduces an enhanced approach based on a Nu-SVR technique to model the MEMS errors. A total of 31,100 samples of static IMU data was collected and divided into training and testing sets. The training sets are used to develop the RBFNN and Nu-SVR models, which were later tested on the independent dataset.

It was found that the Nu-SVR approach performs much better than the RBFNN method in terms of training time and exhibits smaller standard deviation of noises. For the Nu-SVR method, the training time is 10 times faster than the RBFNN method for the gyroscopes. This drastic reduction in the training time is a very beneficial factor for the real-time implementation of the algorithm. Further, our Nu-SVR method reduced the noise standard deviation by 76% when compared with the existing RBFNN approach. The reduction in standard deviations leads to improved navigation solution accuracy. Experimental results under static conditions indicated that Nu-SVR method has successfully enhanced the positional accuracy by 41% in comparison to the RBFNN method. Moreover, in comparison to the generally used GM method, an impressive improvement of 80% is exhibited by the proposed Nu-SVR approach. In future, we will apply our Nu-SVR approach to dynamic data under varied road dynamics and severe environmental conditions.

## Figures and Tables

**Table 1.** Manufacturer specifications for Crista IMU.

**Size**	5.20 cm × 3.93 cm × 2.54 cm		
**Weight**	36.8 g		
**Gyroscope**		**Accelerometer**	
**Range**	±300 °/s	**Range**	±10 g
**In-Run Bias (const.) temp.**	<0.2 °/s	**In-Run Bias**	<0.0245 m/s^2^
**In-Run Bias (vary temp.)**	<0.6 °/s	**In-Run Bias**	<0.500 m/s^2^
**Turn-on to Turn-on**	<0.75 °/s	**Turn-on to Turn-on**	<0.3 m/s^2^
**Alignment Error**	<7.5 °/s (before cal.)	**Alignment Error**	<2.5 m/s^2^
**Bandwidth**	100 Hz	**Bandwidth**	40 Hz
**Noise (ARW)**	3 °/√h	**Noise (VRW)**	0.06 m/s/√h

**Table 2.** Manufacturer and lab calibrated noise (ARW and VRW) values.

	**Calibrated (Const Temp.)**	**Manufacturer (Const Temp.)**	**Calibrated (Varying Temp.)**
**ARW**	3.5 °/√h	3 °/√h	3.50 °/√h
**VRW**	0.10 m/s/√h	0.06 m/s/√h	1.24 m/s/√h

**Table 3.** Gauss Markov parameter for accelerometers and gyroscopes.

**Sensor**	**Correlation Time (T in h)**	**Standard Deviation (σ in m/s^2^_·_°/s)**
**Accelerometer X**	7.38	0.0022
**Accelerometer Y**	7.34	0.0036
**Accelerometer Z**	7.36	0.0022
**Gyroscope X**	5.22	0.0183
**Gyroscope Y**	7.44	0.0195
**Gyroscope Z**	7.71	0.0227

**Table 4.** Performance of gyroscope X with varying number of inputs.

**No. of Inputs (n)**	**S_Raw**	**S_Compensated**	**Time (s)**
**5**	0.2332	0.0552	2.8621
**10**	0.2332	0.0413	1.0500
**15**	0.2332	0.0600	2.2000
**20**	0.2332	0.0767	2.6329

**Table 5.** Standard deviation of the raw and compensated datasets by RBFNN model along with the training time.

**Standard Deviation (°/s, m/s^2^)**	**Gyro X**	**Gyro Y**	**Gyro Z**	**Acc X**	**Acc Y**	**Acc Z**
**Raw**	0.2332	0.2703	0.2311	0.0060	0.0074	0.0079
**Compensated**	0.0413	0.0621	0.0516	0.0035	0.0047	0.0054
**Training time (s)**	1.0500	1.5900	1.3200	0.3035	0.2806	0.2966

**Table 6.** Optimal values of parameters identified for training Nu-SVR.

**Model parameters**	**Gyro X**	**Gyro Y**	**Gyro Z**	**Acc X**	**Acc Y**	**Acc Z**
**C**	10	10	10	10	5	10
**gamma**	0.05	0.10	0.10	5	10	5

**Table 7.** Standard deviation of the raw and compensated datasets.

**Standard Deviation (°/s, m/s^2^)**	**Gyro X**	**Gyro Y**	**Gyro Z**	**Acc X**	**Acc Y**	**Acc Z**
**Raw**	0.2332	0.2703	0.2311	0.0060	0.0074	0.0079
**Compensated**	0.0369	0.0403	0.0352	9.8e-04	0.0011	0.0021
**Training time (s)**	0.1185	0.2647	0.2295	0.1433	0.1967	0.1917
